# Comparison of no-reflow phenomenon in patients with ST-segment- elevation myocardial infarction undergoing primary PCI vs. pharmaco-invasive strategy: a single-centre study

**DOI:** 10.3389/fcvm.2026.1899340

**Published:** 2026-08-13

**Authors:** Ayush Shukla, Awadhesh Kumar Yadav, Akhil Kumar Sharma, Gaurav Kumar Chaudhary, Umesh Chandra Tripathi, Sharad Chandra, Rishi Sethi, Akshyaya Pradhan, Pravesh Vishwakarma, Monika Bhandari, Abhishek Singh

**Affiliations:** Department of Cardiology, King George’s Medical University, Lucknow, India

**Keywords:** no-reflow phenomenon, percutaneous coronary intervention, pharmaco-invasive, ST-elevation myocardial infarction, Cardiovascular diease

## Abstract

**Background:**

The no-reflow phenomenon after percutaneous coronary intervention (PCI) in ST-segment elevation myocardial infarction (STEMI) represents a major barrier to successful reperfusion, leading to adverse outcomes despite epicardial vessel patency. Comparative evidence regarding its incidence and clinical consequences between primary PCI and pharmaco-invasive PCI remains scarce.

**Aim:**

The study aimed to compare the incidence of the no-reflow phenomenon in patients undergoing pharmaco-invasive PCI versus primary PCI, and to evaluate the short-term clinical outcomes of patients with no-reflow at three months post-procedure.

**Methods:**

In this prospective, single centre, observational study, STEMI patients presenting within 24 h of symptom onset were enrolled at a tertiary care centre. Patients experiencing no-reflow were categorized into Group A (primary PCI; *n* = 92) and Group B (pharmaco-invasive PCI; *n* = 192). Baseline demographics, comorbidities, angiographic characteristics, and outcomes were analysed. The primary outcome was the incidence of major adverse cardiac events at three months.

**Results:**

Among 857 patients undergoing primary PCI and 2,976 undergoing pharmaco-invasive PCI, the incidence of no-reflow was significantly higher in the primary PCI group (10.7% vs. 6.5%). Patients in Group A were older (58.9 ± 13.6 vs. 54.4 ± 11.4 years; *P* = 0.004) and more frequently diabetic (55.4% vs. 41.6%; *P* = 0.029), whereas hypertension was more prevalent in Group B (42.7% vs. 13.0%; *P* < 0.001). Baseline TIMI 0 flow was more common in Group A (79.3% vs. 24.5%; *P* < 0.001). After PCI, final TIMI 3 flow rates were comparable (85.9% vs. 89.6%; *P* = 0.361). In-hospital complications and three-month outcomes were similar between two groups: death (4.3% vs. 5.2%), revascularization (4.3% vs. 4.7%), and rehospitalization (5.4% vs. 3.1%) (all *P* > 0.05).

**Conclusion:**

No-reflow was more frequent after primary PCI, particularly in older, diabetic, and late-presenting patients with baseline TIMI 0 flow. Both reperfusion strategies achieved comparable short-term clinical outcomes in no-reflow patients. Pharmaco-invasive PCI may mitigate the risk of no-reflow in selected STEMI patients while maintaining equivalent early prognosis.

## Introduction

Primary percutaneous coronary intervention (PCI) is the preferred reperfusion strategy for patients with acute ST-segment elevation myocardial infarction (STEMI), achieving successful reopening of up to 95% of occluded coronary vessels. However, despite the restoration of epicardial vessel patency, optimal myocardial tissue perfusion is not always achieved (1). This reperfusion failure at the microvascular level is complex and multifactorial, involving distal embolization of thrombotic or atheromatous debris, microvascular spasm, capillary plugging by platelets and leukocytes, and ischemia-reperfusion injury (2). Several patient- and lesion-related factors including older age, diabetes mellitus, hypertension, high thrombus burden, longer lesions, delayed reperfusion, and chronic kidney disease, predispose patients to no-reflow (3).

Ensuring timely reperfusion can be challenging, particularly in regions without immediate access to PCI-capable centres. In these situations, pharmaco-invasive therapy, in which fibrinolytics are administered followed by early angiography and PCI either immediately if fibrinolysis fails or within 24 h if successful, serves as an alternative reperfusion strategy for patients with STEMI (4). Despite these advances, no-reflow remains a major challenge, and comparative evidence regarding its incidence and clinical consequences between primary PCI and pharmaco-invasive PCI is limited. Primary objective of the study was to compare the incidence of the no-reflow phenomenon among STEMI patients undergoing primary PCI and pharmaco-invasive PCI. The secondary objective of study was to compare short-term clinical outcomes among patients who developed the no-reflow phenomenon following primary PCI and pharmaco-invasive PCI.

## Materials and methods

### Study design and population

This was a prospective, single-centre, observational study conducted at a tertiary care center in India between July 2023 and December 2024. The study was approved by the Institutional Ethics Committee, and the written informed consent was obtained from all the patients before enrolment. Inclusion criteria were: (i) patients aged ≥18 years, (ii) patients diagnosed with STEMI of <24 h duration, (iii) patients who presented with typical chest pain and ST-segment elevation ≥1 mm in inferior leads or ≥2 mm in anterior chest leads in two contiguous leads, or with new or presumably new left bundle branch block, and (iv) patients who underwent revascularization by primary PCI in the cardiovascular department. Exclusion criteria include patients with non-ST-elevation myocardial infarction (NSTEMI), who underwent coronary artery bypass grafting (CABG), and those who were presented >24 h after symptom onset.

### Sample size

Sample size was estimated based on the expected difference in the incidence of no-reflow between patients undergoing primary PCI and pharmaco-invasive PCI as reported in previous studies. Van Kranenburg M et al. reported no reflow incidence of 7% (5). Using a 95% confidence level and a 5% error margin, the sample size was determined using the following formula: *n* = *Z*^2^*P*(1−*P*)/*d*^2^.

Where:

*n* = sample size; *Z* = Z statistic for 95% confidence level = 1.96

*P* = expected prevalence = 0.07

*d* = precision = 0.05.

*n* = 1.96 × 1.96 × 0.07 × 0.93/0.05^2^

Therefore, *n* = 100

### Data collection and procedure

All enrolled patients who underwent PCI were divided into two groups. Patients who developed angiographic no-reflow after primary PCI were enrolled in Group A (*n* = 92), while patients who developed no-reflow following pharmaco-invasive PCI enrolled in Group B (*n* = 192). Group A included 92 patients who were enrolled for no-reflow post primary PCI for STEMI whereas Group B included 192 patients who were enrolled for no-reflow post pharmaco-invasive PCI for STEMI. Baseline patient characteristics, cardiovascular risk factors reported within 24 h of admission, angiographic data, in-hospital outcomes, and follow-up at three months were documented. Coronary angiography was performed as per standard institutional protocols, with at least two orthogonal views obtained for each vessel. The number of coronary lesions was determined using quantitative coronary angiography. In the pharmaco-invasive group, Tenecteplase and Streptokinase was given as per physicians’ discretion for thrombolysis. All patients received dual antiplatelet therapy (aspirin and clopidogrel/prasugrel/ticagrelor) prior to the procedure. After administration of 70 IU/kg of heparin stat, PCI was initiated. PCI was considered unsuccessful if the wire could not be crossed, or the stent could not be passed after balloon angioplasty, or residual stenosis exceeded 20% after stenting.

Patients completed a questionnaire including demographic information (age, sex) and cardiovascular risk factors (diabetes, hypertension, dyslipidemia, smoking). Data was collected on time from symptom onset to PCI, and use of thrombectomy devices. TIMI flow was assessed in the first angiographic view after stenting. The no-reflow phenomenon was diagnosed after exclusion of mechanical causes of impaired flow (residual stenosis, dissection, or coronary spasm). TIMI flow was assessed by the operating interventional cardiologist and an independent observer using standard angiographic criteria. Mechanical causes of reduced blood flow were excluded before labelling.

Laboratory parameters measured at admission included serum creatinine, glucose, white blood cells count, red blood cells count, platelet count, mean platelet volume, and red cell distribution width. If a no-reflow phenomenon occurred during PCI, thrombolysis in myocardial infarction (TIMI) grade was documented. Data collection was performed using electronic records and databases maintained by the interventional cardiology department to track adverse outcomes during hospitalization and follow-up.

### Clinical endpoints and definitions

The primary endpoint was the incidence of major adverse cardiac events (MACE) at three months, consisting of death, recurrent MI, target vessel revascularization (TVR), and CABG. Secondary endpoints included death from any cause, recurrent MI (defined as new symptoms/electrocardiographic (ECG) changes and elevated cardiac markers beyond the upper normal limit), TVR (repeat PCI within the same vessel) and severe bleeding (defined as bleeding academic research consortium type 3 or 5 bleeding).

STEMI was defined as chest pain suggestive of MI lasting approximately 30 min, ST-segment elevation ≥1 mm in inferior leads or ≥2 mm in anterior chest leads in two contiguous leads or new/presumed new left bundle branch block on 12-lead ECG, and elevated cardiac markers (creatine kinase-MB or troponin I/T). Primary PCI was defined as PCI performed within 12 h of symptom onset without prior fibrinolysis. Pharmaco-invasive PCI was defined as routine coronary angiography with PCI performed within 3–24 h following successful fibrinolysis according to current guideline recommendations, while rescue PCI was performed immediately in cases of failed thrombolysis. Time to reperfusion therapy was defined as the time from symptom onset to balloon inflation or fibrinolytic injection. Rescue PCI was defined as the PCI performed due to persistent symptoms or ST elevation with <50% resolution within 90 min of fibrinolysis. Urgent PCI referred to PCI for worsening ischemia, hemodynamic instability, refractory arrhythmias, or recurrent ST elevation. Facilitated PCI was defined as PCI within 3 h following fibrinolysis. The use of thrombus aspiration, glycoprotein IIb/IIIa inhibitors, intracoronary vasodilators, thrombectomy devices, and post-dilatation was left to the operator's discretion and was recorded. PCI-associated delay was the time difference between first medical contact (FMC) to balloon inflation and FMC to fibrinolysis. The no-reflow phenomenon was defined as reopening of the occluded coronary artery with successful stent placement on angiography and/or TIMI flow grades 0, 1, or 2.

### Statistical analysis

Data analysis was done using SPSS Software (version 19.0v, SPSS, Inc., Chicago, IL, USA). Quantitative variables were expressed as mean ± standard deviation (SD), while qualitative variables were presented as frequency and percentage. Categorical variables were analyzed using the Chi-square (*χ*^2^) test or Fisher's exact test (for expected frequency <5). Quantitative variables were analyzed using the independent samples t-test for normally distributed data, or the Mann–Whitney U test for non-normally distributed data. Statistical significance was defined as a *p*-value ≤ 0.05, with a confidence interval of 95%.

## Results

**Figure 1:**
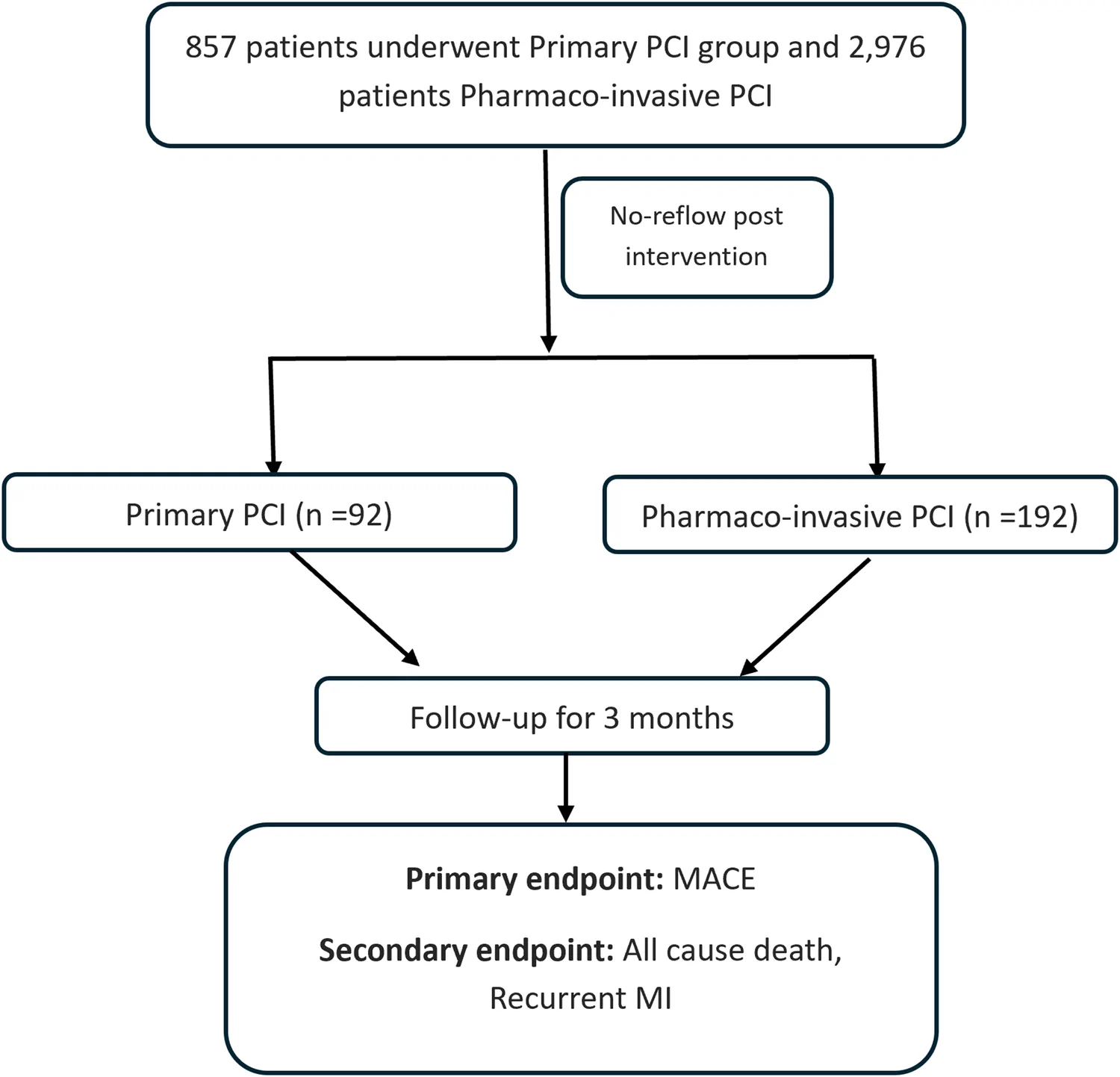
Patient flow diagram.

During study period, a total of 857 patients underwent primary PCI, and 2,976 patients underwent pharmaco-invasive PCI. Among these, the incidence of no-reflow reported in 92 (10.7%) patients in primary PCI (Group A) and in 192 (6.5%) patients in pharmaco-invasive PCI (Group B) as shown in Figure 1.

Majority of the patients were between the age group of 51–60 years. The mean age of the patients in Group A was 58.86 ± 13.56 years and in Group B was 54.35 ± 11.40 years (*P* = 0.004). Males predominated in both groups, with a higher proportion in Group B (88.0%) than in Group A (73.9%). The prevalence of diabetes mellitus was higher in Group A compared to Group B (55.4% vs. 41.6%; *P* = 0.029). Conversely, hypertension was significantly more common in Group B (42.7% vs. 13.0%; *P* < 0.001). CAD was present in 12 (13.0%) patients of Group A and in 38 (19.8%) patients of Group B.

Anterior wall myocardial infarction (AWMI) in Group A was reported in 56 (60.9%) patients while in Group B AWMI was noted in 85 (44.3%) (*P* = 0.008). In contrast, inferior wall myocardial infarction (IWMI) was more frequent in Group B compared to Group A (25.5% vs. 14.1%; *P* = 0.029). Left anterior descending artery (LAD) was the most common culprit artery in Group A and Group B, with no significant difference (56.5% vs. 46.4%; *P* = 0.108). Electrocardiographic findings were comparable between the Groups (*P* = 0.543), indicating a similar distribution of MI locations. Baseline clinical, angiographic and electrocardiographic characteristics are detailed in Table 1.

**Table 1:** Baseline clinical, angiographic and electrocardiographic characteristics.

Parameters	Group A (*n* = 92)	Group B (*n* = 192)	Chi-square/*t*-value	*P*-value
Age group				
≤40	6 (6.5%)	25 (13.0%)	2.702	0.100
41–50	23 (25.0%)	53 (27.6%)	0.215	0.642
51–60	29 (31.5%)	54 (58.1%)	0.347	0.555
61–70	14 (15.2%)	47 (24.5%)	3.163	0.075
71–80	12 (13.0%)	12 (6.2%)	3.710	0.054
>80	8 (8.7%)	1 (0.5%)	13.545	<0.001
Mean age; (years)	58.86 ± 13.56	54.35 ± 11.40	2.930	0.004
Gender				
Male	68 (73.9%)	169 (88.0%)	8.964	0.003
Female	24 (26.1%)	23 (12.0%)		
Diabetes mellitus	51 (55.4%)	80 (41.6%)	4.745	0.029
Hypertension	12 (13.0%)	82 (42.7%)	24.718	<0.001
Smoking	43 (46.7%)	75 (39.1%)	1.509	0.219
Tobacco	43 (46.7%)	71 (37.0%)	2.466	0.116
Coronary artery disease	12 (13.0%)	38 (19.8%)	1.953	0.162
Ejection fraction	44.58 ± 7.05	44.70 ± 8.29	0.121	0.903
HDL (mg/dl)	47.69 ± 21.94	46.71 ± 12.16	0.484	0.629
LDL (mg/dl))	108.98 ± 30.9	102.48 ± 35.10	1.513	0.131
STEMI location				
AWMI	56 (60.9%)	85 (44.3%)	6.855	0.008
IWMI	13 (14.1%)	49 (25.5%)	4.729	0.029
LWMI	8 (8.7%)	23 (12.0%)	0.690	0.406
IWMI + PVMI + RVMI	15 (16.3%)	35 (18.2%)	0.159	0.690
Culprit artery				
LAD	52 (56.5%)	89 (46.4%)	2.572	0.108
RCA	25 (27.2%)	57 (29.7%)	0.191	0.662
LCX	13 (14.1%)	40 (20.8%)	1.841	0.175
LMCA	2 (2.1%)	3 (1.6%)	0.134	0.714
PDA	0 (0.0%)	3 (1.6%)	1.453	0.228

AWMI, anterior wall myocardial infarction; HDL, high-density lipoprotein; IWMI, inferior wall myocardial infarction; LAD, left anterior descending artery; LCX, left circumflex artery; LDL, low-density lipoprotein; LMCA, left main coronary artery; LWMI, lateral wall myocardial infarction; RVMI, right ventricular myocardial infarction; PCI, percutaneous coronary intervention; PDA, posterior descending artery; PWMI, posterior wall myocardial infarction; RCA, right coronary artery; STEMI, ST-segment elevation myocardial infarction.

Around 19 (9.9%) of the patients in pharmaco-invasive group received pre-hospital fibrinolysis while 173 (90.1%) of patients received fibrinolysis in hospital. Similarly, among all thrombosed patients, 20 (10.4%) received Tenecteplase, remaining 172 (89.6%) received streptokinase.

At baseline, TIMI flow grade 0, was markedly more common in Group A (79.3%) compared to Group B (24.5%), indicating a higher incidence of total occlusion at the time of intervention in Group A (*P* < 0.001). Post intervention, TIMI flow grades (0, 1, 2, and 3) were similar between both groups with no statistically significant differences (*P* > 0.05). Use of adjunctive pharmacological agents during PCI was comparable: diltiazem was administered to 12 (13.0%) patients in Group A and 25 (13.0%) in Group B, adenosine to 64 (69.6%) in Group A and 124 (64.6%) in Group B, and nitroglycerin to 16 (17.4%) in Group A and 43 (22.4%) in Group B (*P* = 0.611). Glycoprotein IIb/IIIa inhibitor was used in 16 (17.4%) patients in group A, while 43(22.4%) in Group B (*P* = 0.611). A significantly higher proportion of patients in the Pharmaco-invasive group presented within 3–6 h of symptom onset (30.7% vs. 16.3%; *p* = 0.009). Additionally, more patients in the Primary PCI group presented after 6 h (72.8% vs. 57.3%; *p* = 0.011).

Stent implantation patterns differed between groups. The majority of Group A patients (87.0%) received a single stent, whereas Group B patients had a varied distribution: 52.6% received one stent, 27.1% received two stents, and 18.2% received three stents. A significantly higher proportion of patients presented after >6 h of symptom onset in Group A compared to Group B (72.8% vs. 57.3%; *P* = 0.011). The mean stent diameter was 3.04 ± 0.42 mm and 3.07 ± 0.43 mm in Group A and Group B, respectively (*P* = 0.575). Mean stent length was 27.78 ± 9.30 and 27.36 ± 10.32 mm in Group A and Group B, respectively (*P* = 0.742). Thrombolysis in myocardial infarction flow, pharmacological treatment and stent characteristics are represented in Table 2.

**Table 2:** Thrombolysis in myocardial infarction flow, pharmacological treatment and stent characteristics.

Parameters	Group 1 (*n* = 92)	Group 2 (*n* = 192)	Chi-square/*t*-value	*P*-value
TIMI flow at baseline				
0	73 (79.3%)	47 (24.5%)	76.742	<0.001
1	13 (14.1%)	72 (37.5%)	16.197	<0.001
2	6 (6.5%)	73 (38.0%)	30.734	<0.001
TIMI flow after drug intervention				
0	2 (2.2%)	3 (1.6%)	0.134	0.714
1	4 (4.3%)	6 (3.1%)	0.274	0.601
2	7 (7.6%)	11 (5.7%)	0.370	0.543
3	79 (85.9%)	172 (89.6%)	0.835	0.361
Drug given				
Glycoprotein IIb/IIIa inhibitors	16 (17.4%)	43 (22.4%)	0.983	0.611
Diltiazem	12 (13.0%)	25 (13.0%)		
Adenosine	64 (69.6%)	124 (64.6%)		
Nitro-glycerine	16 (17.4%)	43 (22.4%)		
Number of stents				
1	80 (87.0%)	101 (52.6%)	33.229	<0.001
2	10 (10.9%)	52 (27.1%)		
3	2 (2.2%)	35 (18.2%)		
4	0 (0.0%)	4 (2.1%)		
Window period (hours)				
<3 h	10 (10.9%)	23 (12.0%)	0.075	0.785
3–6 h	15 (16.3%)	59 (30.7%)	6.717	0.009
>6 h	67 (72.8%)	110 (57.3%)	6.392	0.011
Stent diameter (mm)	3.04 ± 0.42	3.07 ± 0.43	1.051	0.575
Stent length (mm)	27.78 ± 9.30	27.36 ± 10.32	0.330	0.742

TIMI, thrombolysis in myocardial infarction.

Following PCI, arrhythmia occurred in 7 (7.6%) patients of Group A and in 15 (7.8%) patients of Group B (*P* = 0.952), congestive heart failure was reported in 15.2% and 14.6% patients in Group A and Group B, respectively. In-hospital mortality was one in Group A and two in Group B. At three-month follow-up, death occurred in 4 (4.3%) patients of Group A and in 10 (5.2%) patients of Group B (*P* = 0.754). Revascularization was noted in 4 (4.3%) patients and in 9 (4.7%) patients of Group A and Group B, respectively (*P* = 0.898). Rehospitalization was reported in 5(5.4%) patients of Group A and in 6 (3.1%) patients of Group B (*P* = 0.345). Complications after percutaneous coronary intervention and at three-month follow-up are illustrated in Table 3.

**Table 3:** Complications after percutaneous coronary intervention and at three-month follow-up.

Complication	Group 1 (*n* = 92)	Group 2 (*n* = 192)	Chi-square/*t*-value	*P*-value
In-hospital complication				
Arrhythmia	7 (7.6%)	15 (7.8%)	0.004	0.952
All-cause mortality	1 (1.1%)	2 (1.0%)	0.001	0.972
Recurrent MI	0 (0%)	0 (0%)	–	–
Severe bleeding (BARC 3 or 5)	0 (0%)	0 (0%)	–	–
Congestive heart failure	14 (15.2%)	28 (14.6%)	0.020	0.888
Hypotension	15 (16.3%)	21 (10.9%)	1.618	0.203
Three-month follow-up				
All-cause mortality	4 (4.3%)	10 (5.2%)	0.098	0.754
Recurrent MI	0 (0%)	0 (0%)	–	–
Target vessel revascularization	4 (4.3%)	9 (4.7%)	0.016	0.898
Severe bleeding (BARC 3 or 5)	0 (0%)	0 (0%)	–	–
Rehospitalization	5 (5.4%)	6 (3.1%)	0.891	0.345

PCI, percutaneous coronary intervention.

## Discussion

The present study compared clinical, angiographic, and short-term outcomes of patients experiencing the no-reflow phenomenon, after two reperfusion strategies for STEMI: primary PCI (Group A) and pharmaco-invasive PCI (Group B). The mean age in Group A was 58.86 ± 13.56 years, and Group B was 54.35 ± 11.40 years (*P* = 0.004). Similarly, Yang L et al., reported age ≥65 years as an independent risk factor for no-reflow (OR = 1.77; 95% CI 1.31–2.38; *P* < 0.001) (3). Our findings reinforce the notion that elderly patients undergoing immediate mechanical reperfusion remain particularly vulnerable to microvascular dysfunction.

With respect to baseline comorbidities, diabetes mellitus was significantly more frequent in the primary PCI group compared with the pharmaco-invasive group (55.4% vs. 41.6%; *P* = 0.029), whereas hypertension was more prevalent in the pharmaco-invasive arm (*P* < 0.001). This pattern suggests that diabetic patients, often with more complex lesions and greater thrombotic burden, may be preferentially directed toward immediate mechanical reperfusion, while hypertensive patients are more commonly managed within the pharmaco-invasive pathway. Similarly, a previous study by Dziewierz A et al., showed that diabetic STEMI patients undergoing primary PCI had longer reperfusion delays, worse angiographic profiles, and higher periprocedural mortality compared to non-diabetics (6). Taken together, these findings highlight diabetes as a key driver of lesion complexity and procedural risk in patients treated with primary PCI. In contrast, Lazzeri C et al. found no clear link between hypertension and reperfusion outcomes, underscoring that the higher prevalence of hypertension in our pharmaco-invasive group may reflect practice patterns rather than a true biological effect (7).

The comparative distribution of STEMI locations between the two-groups revealed distinct patterns in infarct territory involvement. AWMI was observed more frequently in Group A, comprising 60.9% patients, whereas it accounted for only 44.3% patients in Group B (*P* = 0.008), suggesting a higher burden of AWMI among patients undergoing immediate mechanical reperfusion. Conversely, IWMI appeared to be more prevalent in Group B compared to Group A (25.5% vs. 14.1%; *P* = 0.029). These findings are consistent with Sethi R et al., who observed a predominance of IWMI among pharmaco-invasive patients in an Indian cohort (8). Despite these differences, mean left ventricular ejection fraction (LVEF) was comparable between the two groups (*P* = 0.903). This suggests that both reperfusion strategies provided similar myocardial salvage, despite the greater proportion of AWMI in the primary PCI group.

Angiographic analysis showed that baseline TIMI flow was significantly worse in the primary PCI group, with 79.3% presenting with TIMI 0 flow compared to only 24.5% in the pharmaco-invasive group. Conversely, TIMI 1–2 flows were more common in the pharmaco-invasive group (37.5% and 38.0%). After intervention, however, TIMI 3 flow was restored in 85.9% of primary PCI patients and 89.6% of pharmaco-invasive patients (*P* > 0.05). These results indicate that although pharmaco-invasive patients often arrived with partial flow due to prior fibrinolysis, both strategies ultimately achieved similar procedural reperfusion success. Although final TIMI 3 flow rates were comparable between the two no-reflow groups, this reflects restoration of epicardial coronary flow and does not necessarily indicate equivalent microvascular perfusion. The temporal profile of presentation differed significantly between groups. A higher proportion of patients in the pharmaco-invasive group (30.7%) presented within 3–6 h of symptom onset compared to the primary PCI group (16.3%; *P* = 0.009), whereas late presentation beyond 6 h was more frequent in the primary PCI group (72.8% vs. 57.3%; *P* = 0.011). Ultra-early presentation (<3 h) was low in both Group A (10.9%) and Group B (12.0%) (*P* = 0.785). This suggests that pharmaco-invasive protocols may facilitate earlier hospital arrival through pre-hospital fibrinolysis and streamlined referral pathways. Our findings are in line with Paredes-Paucar C et al., who reported that primary PCI was independently associated with higher no-reflow risk compared with pharmaco-invasive approaches in STEMI patients (9). Prolonged ischemia time and baseline TIMI 0 flow likely explain the greater no-reflow burden in our late-presenting primary PCI cohort. Consistent to our findings, Pradhan A et al. demonstrated that deferred PCI in patients with high thrombus burden reduced no-reflow risk by allowing gradual reperfusion (10). Collectively, these studies support the concept that staged or pharmacologically facilitated reperfusion strategies may mitigate microvascular obstruction (9, 10).

In terms of in-hospital complications, rates of arrhythmia, death, congestive heart failure, and hypotension were comparable between the groups, with no statistically significant differences (all *P* > 0.05), aligning with observations by Mentias A et al. (11) At 3-month follow-up, both groups demonstrated similar outcomes. Mortality was slightly higher in the pharmaco-invasive group (5.2% vs. 4.3%), repeat revascularization rates were nearly identical (4.7% vs. 4.3%), and rehospitalization was marginally more frequent in the primary PCI group (5.4% vs. 3.1%). None of these differences reached statistical significance, indicating that both reperfusion strategies achieved broadly comparable short-term clinical outcomes. Our results suggest that pharmaco-invasive PCI offers outcomes comparable to primary PCI in real-world STEMI patients, while potentially reducing the risk of no-reflow in late presenters.

### Study limitations

This study has several limitations. First, this study was conducted at a single centre in India, which may limit the generalizability of the findings to other populations or practice settings. Second, the sample size, although adequate for comparative analysis, remains relatively small, and larger multicentre studies are needed to validate these observations. Third, the observational design precludes establishing causal relationships, and potential confounding factors such as variations in operator technique, pharmacotherapy, and referral delays could not be fully controlled. The comparisons between the no-reflow groups were based on unadjusted analyses. Given the observational study design and baseline differences (including age, diabetes mellitus, and initial TIMI flow) between the groups, residual confounding cannot be excluded. Finally, outcomes were assessed only up to three months, and longer-term follow-up is necessary to determine whether the differences in no-reflow incidence translate into significant prognostic implications over time.

## Conclusion

In this study, no-reflow occurred more frequently following primary PCI compared with pharmaco-invasive PCI in STEMI patients, particularly among late presenters and those with baseline TIMI 0 flow. Among patients who developed the no-reflow phenomenon, short-term clinical outcomes were comparable between the Primary PCI and pharmaco-invasive groups despite differences in the incidence of no-reflow. These findings suggest that pharmaco-invasive PCI may reduce the risk of no-reflow in selected patients with comparable short-term clinical outcomes among patients who developed no-reflow. However, given the comparable rates of final TIMI 3 flow and baseline differences between groups, these findings should be interpreted cautiously. Further larger, multicentre studies with longer follow-up are warranted to optimize patient selection and reperfusion strategies in STEMI.

## Data Availability

The raw data supporting the conclusions of this article will be made available by the authors, without undue reservation.
